# Dynamic study of the finger interphalangeal joint volar plate—motion analysis with magnetic resonance cinematography and histologic comparison

**DOI:** 10.1007/s00256-023-04288-6

**Published:** 2023-02-10

**Authors:** Christoph Lutter, Stefanie Kuerten, Carol Geppert, Wolfram Mittelmeier, Annett Klinder, Stephan Söder, Frank Römer, Michael Uder, Rolf Janka, Thomas Bayer

**Affiliations:** 1 Department of Orthopedics, University Medical Center, 18057 Rostock, Germany; 2 Department of Anatomy and Cell Biology, Friedrich Alexander University, 91054 Erlangen-Nuremberg, Germany; 3 Institute of Neuroanatomy, Medical Faculty, University of Bonn, 53115 Bonn, Germany; 4 Department of Pathology, Friedrich Alexander University, 91054 Erlangen-Nuremberg, Germany; 5 Institute of Pathology, Klinikum Fürth, 90762 Fürth, Germany; 6 Institute of Radiology, Friedrich Alexander University, 91054 Erlangen-Nuremberg, Germany; 7 Department of Radiology and Neuroradiology, Klinikum Fürth, 90762 Fürth, Germany

**Keywords:** MRI, Injury, Dislocation, Microsurgery, Ultrasound

## Abstract

**Objective:**

We aimed to further improve knowledge about volar plate (VP) motion of the finger proximal interphalangeal joint (PIP), by analyzing the dynamic VP shape during a full range of finger flexion using magnetic resonance cinematography of the fingers (MRCF), and to compare the results with anatomical cross sections from cadaver specimens.

**Materials and methods:**

The dynamic sagittal VP shape was visualized with MRCF in a total number of 23 healthy volunteers. The length, angle, and thickness as well as the contact length of the VP to the PIP joint base were measured. Statistical analysis included *t*-test or rank-sum testing. Anatomical cross sections with differing degrees of PIP joint flexion were obtained from 12 cadaver specimens (fingers) for comparison.

**Results:**

Significant positive correlations between PIP joint flexion angle and VP area, length, depth and the VP contact length were found. This matched histologically to fiber rearrangements especially within the loose third VP layer.

**Conclusion:**

Our study analyzed the full range of motion dynamic VP shape of the PIP joint using MRCF. This contributes to a more precise understanding of the complex interaction of the VP with the PIP joint and may facilitate evaluation of clinical cases such as VP avulsion or pulley rupture.

**Supplementary Information:**

The online version contains supplementary material available at 10.1007/s00256-023-04288-6.

## Introduction

**Fig. 1:**
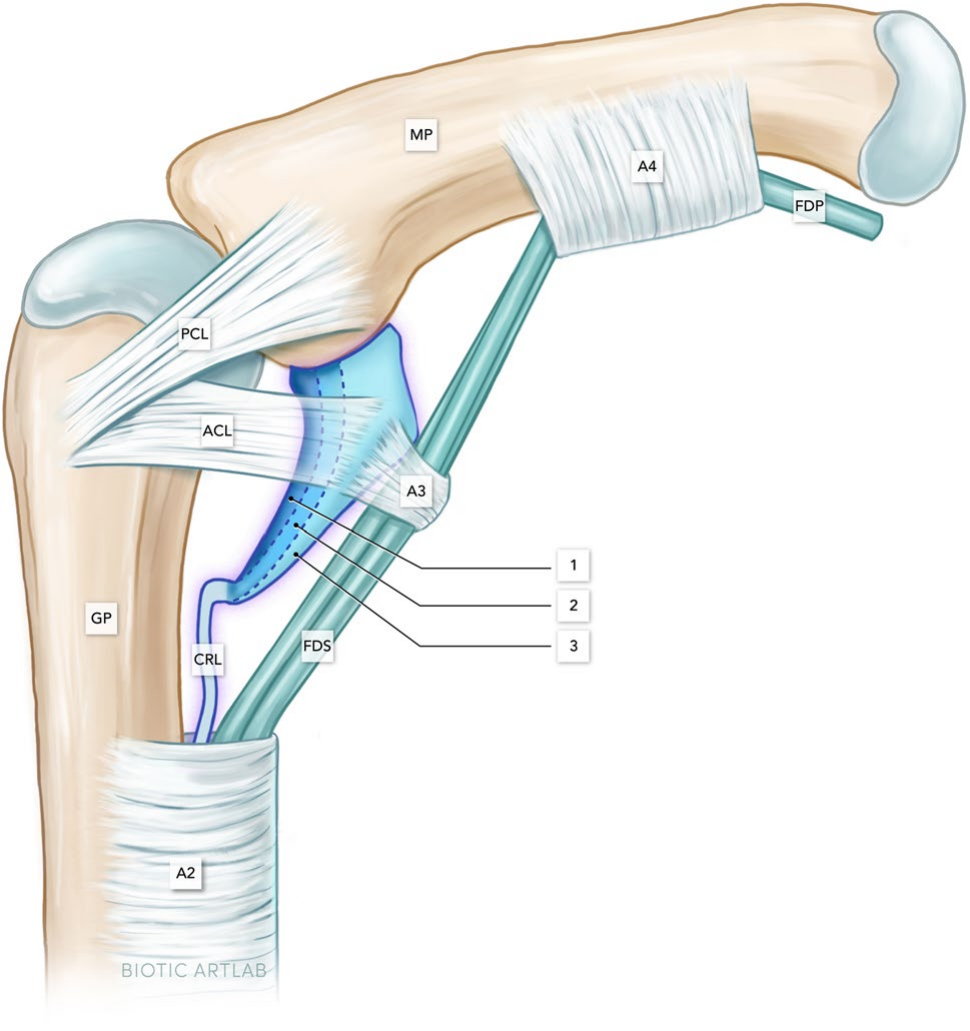
Microanatomical illustration of the PIP joint and volar Plate (VP) at 60° flexion. The VP consists of a cartilaginous part (i.e., VP body), which contains 3 layers and a membranous part, which contains the check rein ligaments: PCL, proper collateral ligament; ACL, accessory collateral ligament; FDP, flexor digitorum profundus tendon; FDS, flexor digitorum superficialis tendon; 1, 1st volar plate layer; 2, 2nd volar plate layer, from which the check rein ligaments arise; 3, 3rd volar plate layer; A2, A2 pulley; A3, A3 pulley, A4, A4 pulley; GP, ground phalanx; MP, middle phalanx; CRL, check rein ligament

The volar plate (VP) of the proximal interphalangeal (PIP) joint has utmost importance for the physiological interaction of flexor tendons and joints during finger flexion [1, 2]. It ensures a stabilization against overextension, lateral displacement, and torsional forces and increases the moment of flexion [3–5]. In addition, it forms a mobile origin of the flexor tendon A3 pulley [6, 7], which is crucial for force transmission from the flexor tendons to the fingers (Fig. 1).

The VP integrity may be affected in various everyday injuries such as PIP joint distortion, avulsion, or pulley rupture. Numerous conservative and surgical techniques have been described, including pulley graft and direct pulley suturing for pulley rupture and direct VP suturing with or without the use of bone anchors and the tenodesis technique with flexor digitalis superficialis (FDS) for VP avulsion [8–11]. However, the outcomes of the available procedures are not always fully predictable and satisfactory [8–11], and cases may degenerate into chronic conditions such as flexion contracture and function loss. This justifies studies aiming for a more precise understanding of the physiological VP functionality, which could possibly also be used for improved diagnostics of such injuries.

Histologically, the VP consists of a membranous proximal more flexible part and a cartilaginous thicker and stiffer distal part, which is also referred to as the VP body [4, 6]. Several authors have studied the dynamic range of the VP, which was attributed to its complex fibrocartilaginous composure ensuring different biomechanical requirements [2, 6, 7, 12]. Watanabe et al. reported a histologic architecture with a three-layer model (Fig. 1) inside the VP body with perpendicular fibers resembling a basket weave [6]. It was presumed that this fiber composure would contribute to a better stabilization during deformation of the VP when fingers are flexed [13–16].

Studies analyzing the dynamic VP shape in healthy volunteers during a full range of motion with systematic admission of metric parameters are pending, because no imaging modality could provide the specific requirements for such an analysis. Recently, functional magnetic resonance cinematography of the fingers (MRCF) became available as a new imaging technique [17]. In comparison to functional ultrasound (US), it is independent from the individual skills of the performing physician, and it allows a dynamic visualization of the finger anatomy in the entire range of motion in real time [17]. Therefore, the purpose of this study was to analyze the dynamic VP shape during finger motion using MRCF and to associate the results to the histological findings from cross sections from cadaver specimens.

## Materials and methods

### Study population

A total number of twenty-three consecutive healthy volunteers (male:female = 17:6; mean age 27.3 ± 5 years) were enrolled between May 2017 and July 2018. Four index fingers, 12 middle fingers, and 7 ring fingers were analyzed (one finger from each volunteer). Selection of the examined finger was by random. Exclusion criteria were any history of acute or overuse injuries on the examined hand and prior surgical procedures on the examined upper extremity.

### Imaging

**Fig. 2:**
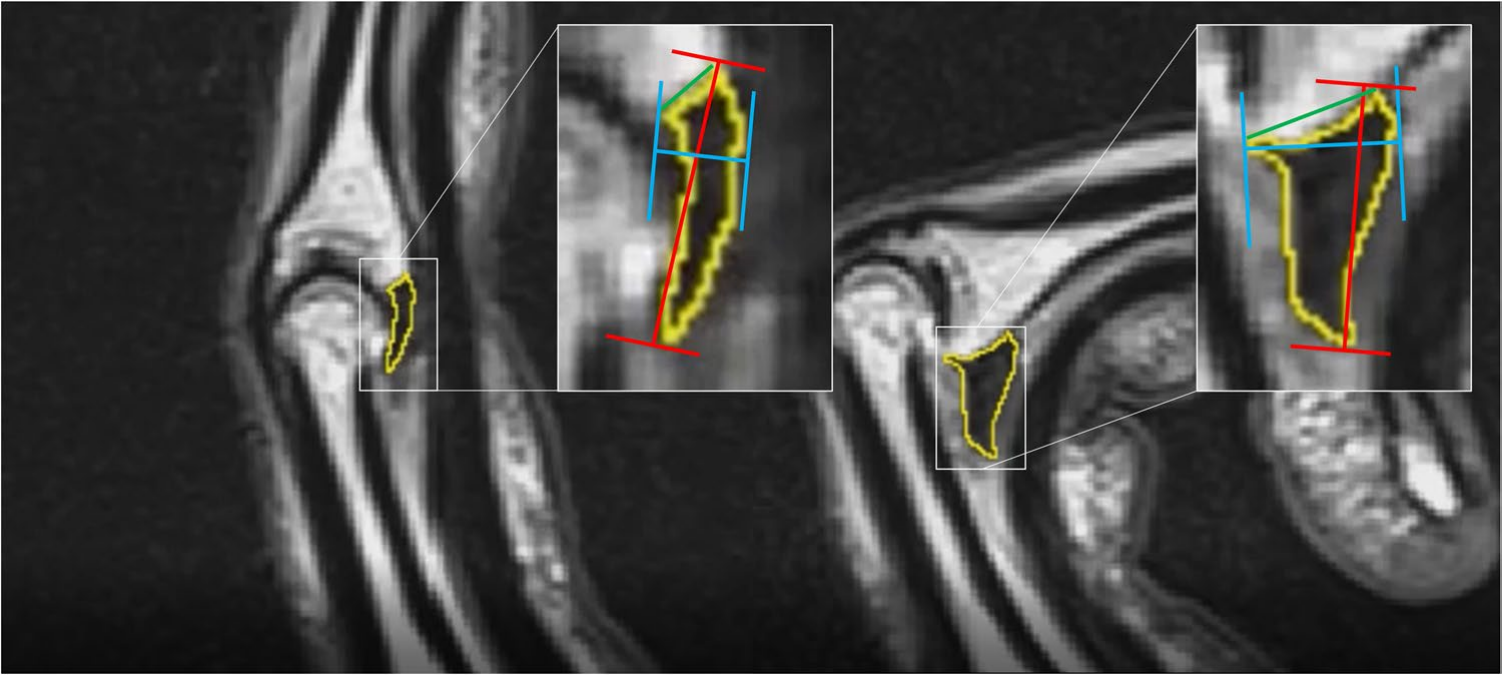
Exemplary image analysis with metric measurements: area measurement (yellow). Measurement of Feret max (length, red) and Feret min (depth, blue) corresponding to slide gauge definition of the corresponding VP length and VP depth. The green line defines the length measurement of the contact area of the VP on the base of the middle phalanx

**Fig. 3:**
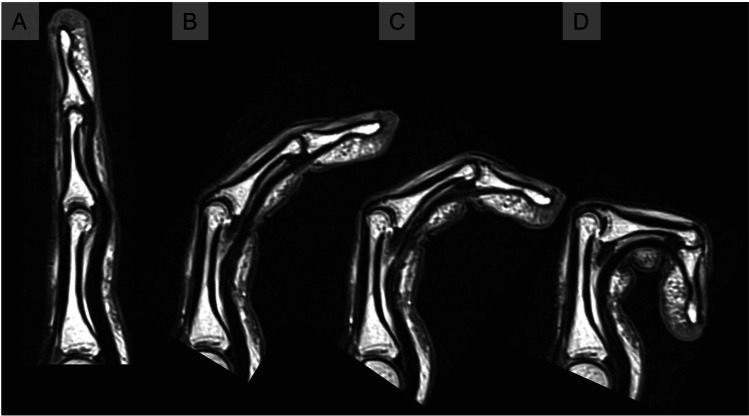
Midsagittal MRCF images of a middle finger during active flexion (**A** full extension, **B** 30° flexion, **C** 60° flexion, **D** 100° flexion)

Every participant was examined according to full range of motion in finger joints. A 3-Tesla (T) whole-body scanner (Magnetom Skyra, Siemens Healthineers, Erlangen, Germany) with a maximum gradient amplitude of 45 mT/m and a rise time of 225 μs was used. A foot and ankle 8-channel phased array receiving coil (8-channel foot and ankle coil, Siemens Healthineers, Erlangen, Germany) was applied to allow space for the finger movement. A custom-made wooden fingergating device with two individually adjustable sidewalls for finger width adjustment was used as described before [17]. Each individual was advised to repetitively extend and flex the examined finger during examination in a full range of motion as slowly as possible without any force applied on the fingertips as described before [17]. A cinematographic real-time T2-weighted TRUFI BEAT® (Syngo BEAT®, Siemens, Erlangen, Germany) sequence and an incremental dynamic time optimized proton density (PD)-weighted turbo spin echo (TSE) sequence were acquired in a sagittal plane as previously published [17]. Frame rates were 59/min and 3/min; pixel size was 0.78 × 0.78 mm and 0.45 × 0.56 mm, respectively. The slice thickness was 3 mm for the BEAT and 2 mm for the PD sequences. Images were then analyzed by an experienced musculoskeletal radiologist (T.B.) and an orthopedic surgeon (C.L.) on a commercially available picture archiving and communication system monitor (PACS SyngoPlaza, Siemens Healthineers, Erlangen, Germany) to confirm the integrity of finger anatomy and evaluate image quality. Only datasets without relevant imaging artifacts were considered for further metrical investigations. All measurements were performed by the two researchers in consensus in a centered sagittal plane as described in previous studies [13, 18, 19] (Figs. 2 and 3; Video).

### Image analysis

Images were then exported as tagged image file format sequences (.tiff files) and imported in Image-J software (Fiji ImageJ 1.52b, https://imagej.nih.gov/ij/), scaled according to the dimensions of the receiving coil, cropped, and finally, magnified (5 ×). Index, middle, and ring finger images were pooled. The analysis included all images of the first complete movement from extension to flexion for each individual. For each image, the flexion angle in the PIP joint was measured with the Fiji ImageJ *Angle Tool*. Therefore, both the proximal and the middle phalanx were longitudinally marked from the center of the proximal joint line to the center of the distal joint line. The angle between these two lines was considered the PIP joint flexion. Images were separated into 12 ten-degree bins according to their PIP joint flexion angle from 0 to 9.99°, 10 to 19.99°, 20 to 29.99°, 30 to 39.99°, …., 110 to119.99°. The corresponding labeling of the bins was for simplicity “ < 10°,” “ < 20°,” “ < 30°,” …, “ < 120°.”

The hypointense volar plate body was outlined with a pen-controlled trackpad; the margin was thereby defined as a relatively abrupt change in intensity to the more hyperintense surrounding connective tissue (Fig. 2). The following parameters were recorded: *Area*, *Feret max (length)*, and *Feret min (depth)*. Feret diameters (caliper) were measured automatically via ImageJ (applying two parallel lines to the edges of the volar plate to measure their distance) (Fig. 2). Feret diameters are length measurements corresponding to the measurement by a slide gauge (slide gauge principle). *Feret min* thereby indicates the smallest possible Feret diameter (i.e., VP depth), while the *Feret max* measures the largest distance between the imaginary lines (i.e., VP length) [20]. The length of the contact area of the volar plate on the base of the middle phalanx (i.e., translation of the VP body relative to the volar lip of the middle phalanx base [13, 18, 19]) was measured as described previously (Fig. 2).

### Histopathology

To compare MRCF findings with histology and to examine a possible relationship with the microstructural 3-layer model as described by Watanabe et al. [6], we prepared additional histological slides obtained from post mortem fingers in the corresponding joint positions. 12 fresh frozen finger specimens (index, middle, and ring finger of 2 left hands, 2 right hands from a total of 2 male cadavers, 39 and 54 years at time of death) were harvested. After thawing, the fingers were randomized into angular positions (one finger at each PIP flexion angle: 0°, 10°, … °, 120°) representing the full range of motion in 10° steps and held in the corresponding position using cable ties. Subsequently, the specimens were frozen again and then cut in sagittal plane using a band saw and transferred for histological processing. Hematoxylin and eosin staining was then performed using standard techniques. Scanning and digitalization of the slides was performed using dedicated software (Case Viewer, 3DHISTECH, Budapest, Hungary). A consensus image reading was performed by defining three VP fiber layers for associating each flexion angle with MRCF by two researchers (CL, TB). Therefore, reference lines were drawn along longitudinally running dense fibers, which had a bony Sharpey fiber insertion. These fibers represented the inner (second) VP layer. The first VP layer was defined at the joint side beyond the most dorsal reference line and contended loose synovial tissue. The third VP layer was defined at the tendon side beyond the most volar reference line and contended perpendicular running loose fibers. The connexions with the checkrein ligaments (second VP layer) and the collateral ligaments (third VP layer) could not be adequately visualized in the sagittal plane due to their lateral position. Distance or angle measurements were omitted due to the shrinking that occurs during processing and the limited number of specimens.

### Statistical analysis

The collected data were analyzed using SPSS Statistics Package Version 27 (IBM Corp., New York, USA). Correlation analyses between the individual flexion angles (before grouping into the 12 ten-degree bins) and the corresponding measured parameters of volar plate area, Feret max, Feret min, and the volar plate contact length were performed by Spearman’s rank correlation test as the measured data were not normally distributed. Additionally, the measured data were assigned to 12 ten-degree bins (PIP joint flexion groups) and allocated to each participant. Descriptive statistics of these data were calculated, and the continuous variables are displayed as mean values and standard deviations (SD), unless stated otherwise. Changes in the measurements due to increased flexion were determined by comparing the PIP joint flexion groups in a repeated-measures general linear model. Missing values in the ten-degree bins of individual participants were calculated by multiple imputation to allow for repeated measurement testing. Sphericity was checked with Mauchly’s sphericity test. If sphericity was not established, lack of sphericity was adjusted by the Greenhouse–Geisser correction. Post hoc tests to allow pairwise comparison between the PIP joint flexion groups were performed with Bonferroni’s correction. Extended fingers (PIP joint flexion group < 10° [= base line]) were considered as control group. *P* values < 0.05 were considered statistically significant.

## Results

### Imaging measurements

A total number *N* = 421 images corresponded to the first full extension–flexion movement of all participants (18.3 ± 8 images per volunteer). All datasets (*N* = 421/421) were free of relevant artifacts and could be used for analysis. Overall, a total number of 2105 individual measurements were performed. Within the twelve groups of PIP joint flexion angles, the average number of images was 35.1 ± 8.7. Table 1 shows the results of individual measurements independent from their allocation to the participants for area, length, depth, and contact length from 0 to 120° flexion. Statistical analysis revealed significant positive correlations between flexion angle and VP area, VP length, VP depth, and the VP contact length, respectively (Table 2). While there was a significant correlation between flexion angle and length, the degree of correlation was very weak. However, all three other measurements showed strong correlations.

**Table 1:** Average measurements of volar plate area (mm^2^), length (mm), depth (mm), and contact length (mm) from 0 to 120° flexion

Flexion degree	Volar plate area	Volar plate length	Volar plate depth	Volar plate contact length
< 10°	10.35 ± 2.13	7.01 ± 0.58	2.48 ± 0.48	1.63 ± 0.35
< 20°	10.12 ± 1.52	6.75 ± 0.80	2.45 ± 0.36	1.63 ± 0.44
< 30°	11.57 ± 1.93	6.86 ± 0.85	2.61 ± 0.42	1.62 ± 0.41
< 40°	12.17 ± 2.43	6.89 ± 0.98	2.88 ± 0.47	1.71 ± 0.44
< 50°	13.74 ± 2.68	6.94 ± 0.82	3.13 ± 0.53	1.73 ± 0.42
< 60°	15.84 ± 3.54	6.87 ± 0.78	3.61 ± 0.60	1.92 ± 0.55
< 70°	17.57 ± 3.76	6.87 ± 0.98	3.97 ± 0.60	2.22 ± 0.70
< 80°	18.91 ± 4.61	7.36 ± 0.96	4.18 ± 0.63	2.36 ± 0.63
< 90°	20.05 ± 4.40	7.52 ± 0.78	4.46 ± 0.64	2.93 ± 0.55
< 100°	20.25 ± 4.92	7.58 ± 1.14	4.40 ± 0.68	3.40 ± 0.58
< 110°	18.84 ± 4.90	7.53 ± 1.26	4.28 ± 0.70	3.73 ± 0.64
< 120°	16.26 ± 4.42	6.98 ± 1.03	4.18 ± 0.78	3.68 ± 0.62

**Table 2:** Correlation of volar plate area, length, depth, and contact length with PIP joint flexion angle (Spearman’s rank correlation coefficient)

		Volar plate area	Volar plate length	Volar plate depth	Volar plate contact length
Flexion angle	*R*	0.616**	0.160**	0.713**	0.707**
	*P*	< 0.001	0.001	< 0.001	< 0.001

### Volar plate area and shape

**Fig. 4:**
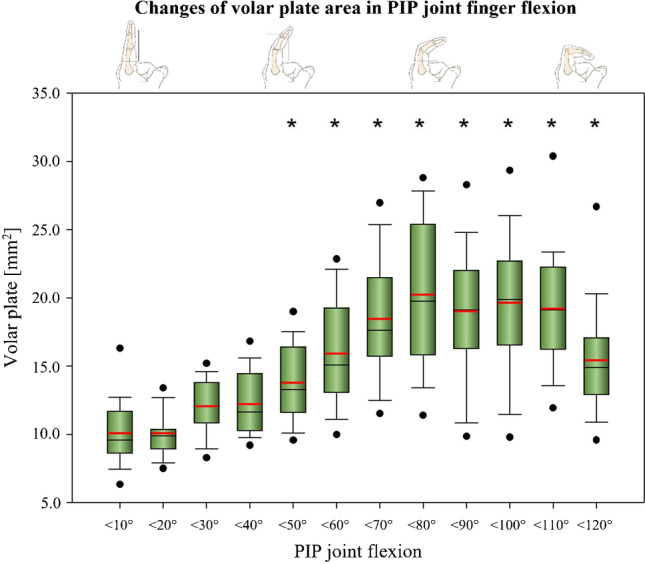
Changes of volar plate area in PIP joint finger flexion. *Significant difference compared with full extension (< 10°). The volar plate area is significantly larger in flexion angles of the groups < 50° and higher

The area covered by the volar plate in a mid-sagittal plain in various PIP joint flexions is presented in Fig. 4. The highest VP area was measured in the group of < 80° of PIP joint flexion. A significantly larger area covered by the VP was found in all groups > 40° PIP joint flexion as compared to base line (full extension).

During flexion, the shape of the VP changed. During this process, it formed various characteristic shapes, which merge smoothly into another. In the sagittal plane evaluated here, the structure assumes an elongated and thin comma shape in extension (Fig. 1A). Up to about 45° of flexion, it retains this comma shape, but loses length and experiences an increase in thickness. Between 45° and max. flexion, the VP then increasingly takes on the shape of a shark fin (Fig. 1D). Throughout the flexion of the finger, the location of the VP contact changes from the proximal end of the phalanx to the volar surface of the bone (see below: VP contact length). An exemplary cinematographic analysis movie is given as supplemental material (Video).

### Volar plate length and depth

**Fig. 5:**
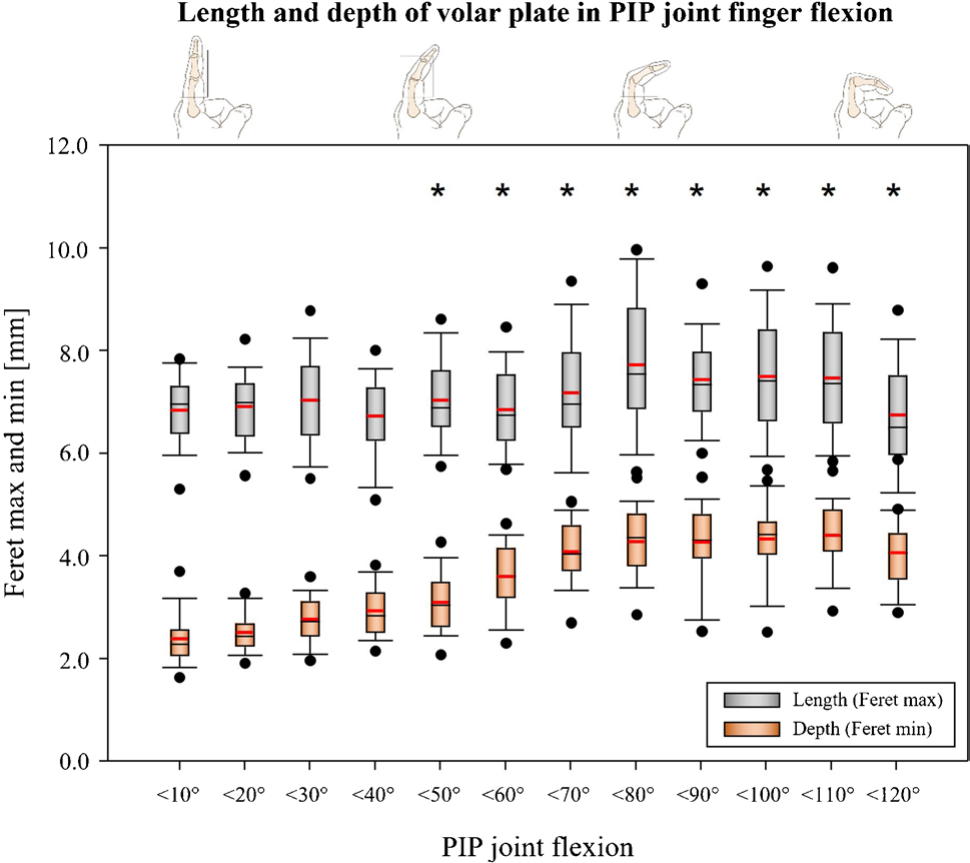
Length and depth of volar plate in PIP joint finger flexion. *Significant difference of depth compared with depth in full extension (< 10°). The volar plate depth is significantly greater in flexion angles of the groups < 50° and higher

Figure 5 shows the *length* and *depth* distribution of the VP as a function of the flexion position of the PIP joint. Length averages 6.8 ± 0.7 mm and depth averages 2.4 ± 0.5 mm when the finger is extended (< 10° PIP joint flexion). Length was found to remain largely constant throughout the range of motion while depth increased steadily to about 80° of flexion. Above 50 degrees of flexion, depth was found to be significantly larger compared to depth in extension (Fig. 3).

### Volar plate contact length

**Fig. 6:**
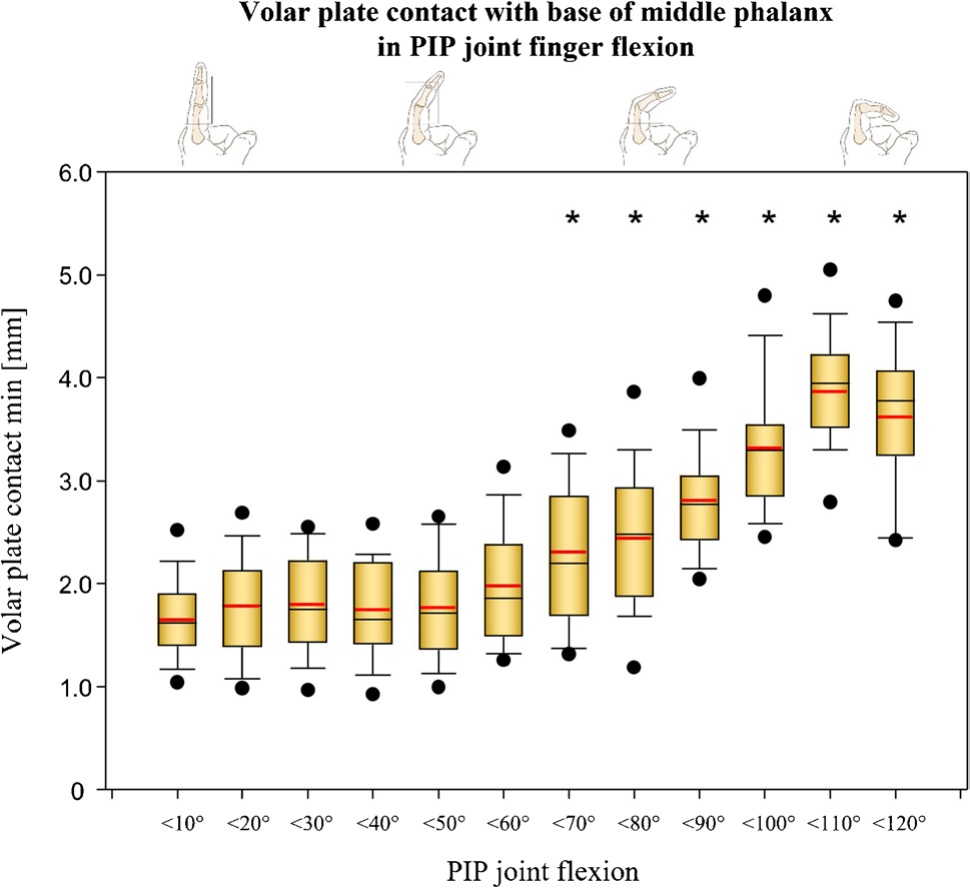
Volar plate contact with base of middle phalanx in PIP joint finger flexion. *Significant difference compared with full extension (< 10°). The volar plate contact with the base of the middle phalanx is significantly greater in flexion angles of the groups < 70° and higher

The length at which the distal end of the VP contacts the base of the middle phalanx changed significantly over the flexion process of the finger. While during extension, an attachment of an average length of 1.6 ± 0.4 mm was detected, this contact length increased continuously during flexion up to a maximum length of 3.9 ± 0.5 mm at 110° flexion in the PIP joint. For all specimens with a PIP joint angle position > 70°, significantly longer contact areas were observed than with an extended finger position (< 10° flexion) (Fig. 6).

### Histological comparison

**Fig. 7:**
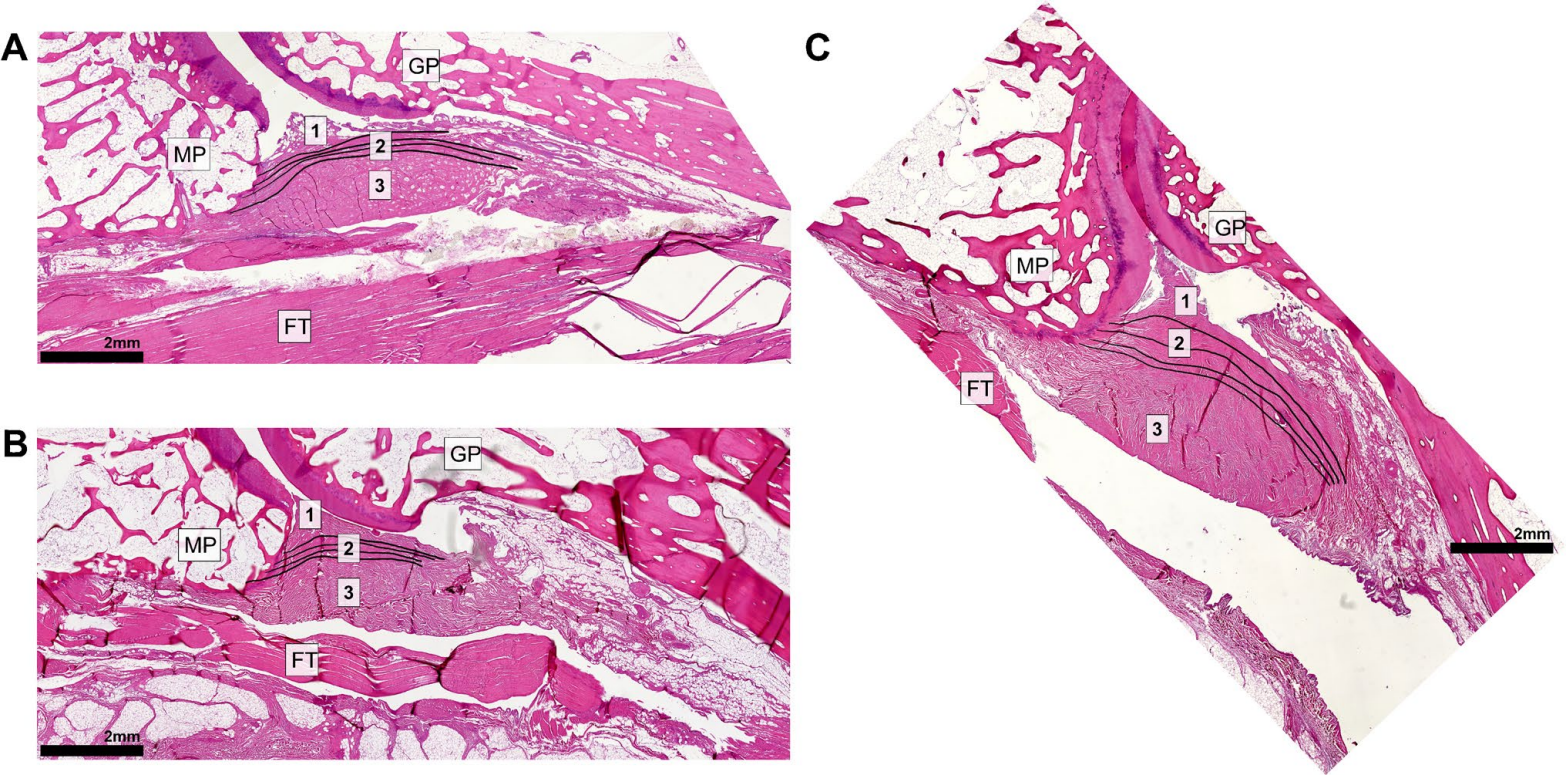
Histological images of fingers in different flexion positions (**A** middle finger, full extension; **B** index, 30° flexion; **C** middle finger, 60° flexion). GP, proximal phalanges; MP, intermediate phalanges; FT, flexor digitorum tendon; 1–3, VP layers 1–3. Note the increasing angle and thickness as well as the increasing contact length of the VP relative to the middle phalanx bases during the higher PIP flexion positions. This correlated especially to the third fiber layer, which was mostly affected by these shape changes during higher flexion

The consensus histopathological reader analysis confirmed a VP shape similar found to that in MRCF in all available slides. The analysis of fiber layers showed that the third VP layer mostly contributed to the shape changes, especially in higher flexion angles. This third layer experienced a special increase of thickness, area, and contact to the middle phalanx, whereas the first and second fiber layers remained relatively constant throughout the increasing degrees of flexion (Fig. 7).

## Discussion

Using MRCF as new imaging approach in a collective of healthy volunteers enabled metric analysis of the entire dynamic VP shape with a full freedom of joint movement for the first time.

**Fig. 8:**
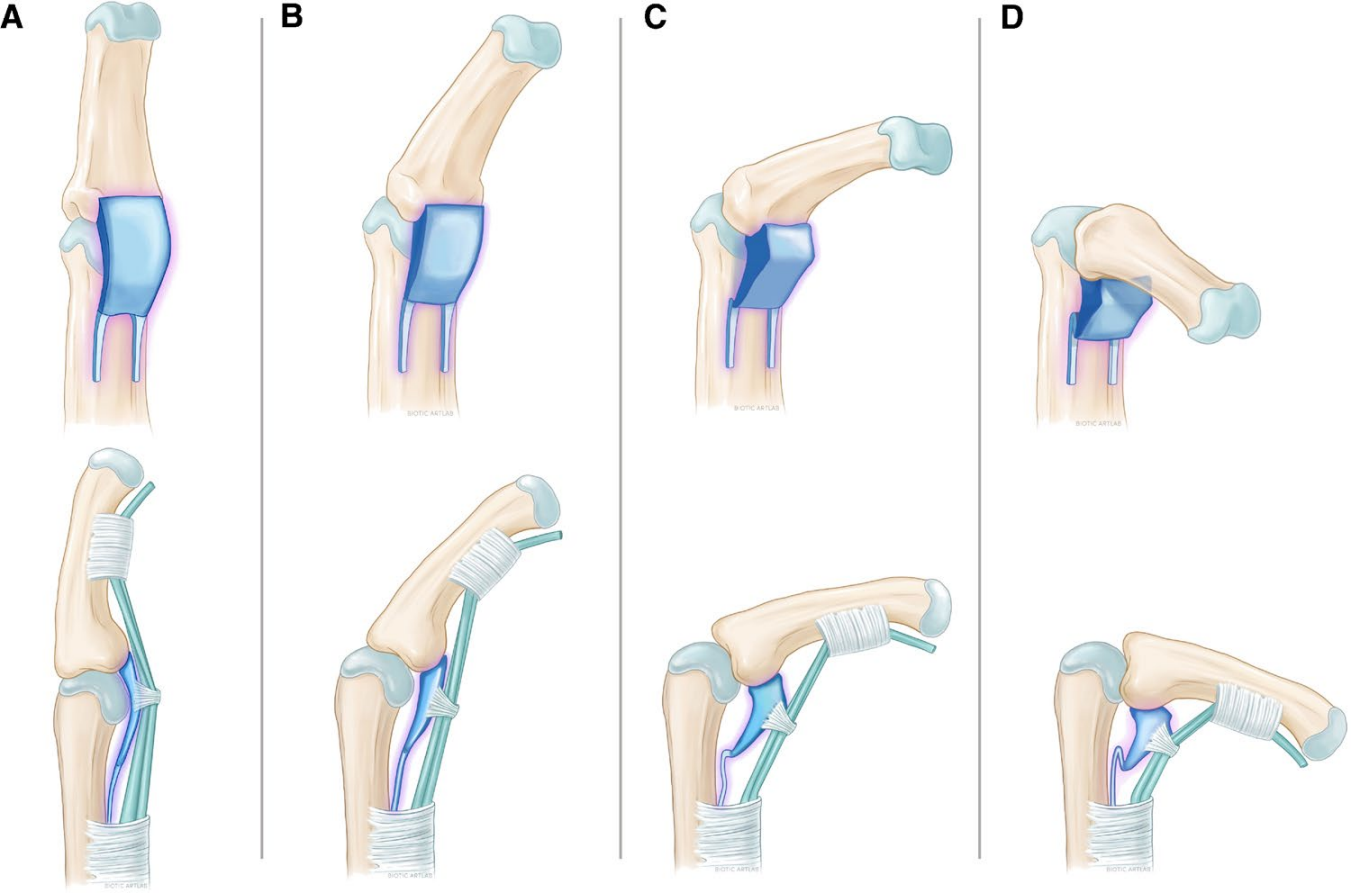
Schematic drawing of the sagittal shape representing the presumed interaction of the VP complex with the adjacent tendons, bones, and pulleys during finger flexion (**A** full extension, **B** 30° flexion, **C** 60° flexion, **D** 100° flexion)

For the lower degrees of flexion, our study confirmed findings of a study of Saito and Suzuki who described a sequence of thickening, bulging, and protrusion of the VP during flexion from 0 to 45° of flexion [16]. Particularly, the elevation phase mentioned in their study was excellently reproduced by MRCF [16]. Based on the results of previous studies, it was expected that there would be extensive changes in VP shape with higher finger flexion. With the present MRCF setup, we were able to monitor this shape rearrangement independently for different metric parameters investigated at different finger flexion positions. We registered a statistically significant increase in the sagittal VP cross sectional area when fingers were flexed beyond the 50° position. Additionally, it was noted that the VP depth increased beyond the 50° position. In contrast to that, our statistical analysis confirmed an increase of contact for the VP with the middle phalanx base if fingers were flexed further beyond 70°. Considering the described complex physiological VP shape rearrangement, we believe that an MRCF analysis adds valuable knowledge with sufficient precision for future study trials. Our analysis contributed to develop a schematic drawing of the sagittal VP shape as shown in Fig. 8.

Although a true anatomical MRCF-correlation was technically not possible, the VP shape found in cadaver specimens matched to the in vivo MRCF findings in different flexion positions. During higher degrees of flexion, we found major ex vivo VP shape changes mainly inside the third fiber layer of the VP as described by Watanabe et al. [6]. The ex vivo examinations support the hypothesis, that especially the third layer, contending loose perpendicular running fibers, contributes to the increase of area, depth, and contact length. We noted that the first and second layer contending synovial tissue and dense longitudinal running fibers respectively had relatively constant fiber courses. In this respect, it is likely that the VP area changes found with MRCF are mainly the result of fiber deformations within the third VP layer containing loose perpendicular running fibers.

Our study results suggest a key role of the VP in the complex interplay between different stabilizing requirements for the PIP joint depending on finger flexion. For PIP joint extension, the function of stabilization in the anteroposterior plane comes to the fore. The elongated flat shape of the VP in extension with straight dense histological fibers in the mid layer of the VP is most likely the main correlate for hyperextension protection. In contrast, during increasing finger flexion, requirements for maintaining force transmission, stabilization of the PIP joint with the collateral ligaments in the lateral plane, and ultimately undisturbed tendon gliding during force transmission probably come to the fore. The change in position of the VP is undoubtfully necessary to prevent intercalation between the proximal phalanx condyle and middle phalanx, especially at higher degrees of flexion beyond 70° [13]. This movement is probably dependent from force transferred to the VP via the flexor tendons and the A3 Pulley [13]. Furthermore, the change in position and shape as well as change of fiber courses in the third VP layer in the degrees of flexion above 50–70 degrees probably supports stabilization additionally in lateral PIP dimension by connection with the accessory collateral ligaments and in all probability supports the guidance of the flexor tendons and force transmission via the A3 pulley ligament.

It is noteworthy that relatively few studies have investigated the complex microanatomical structure and physiological biomechanics of the VP and even today there may still be incomplete knowledge about the VP function. It was not until 1967 that a specific study examined the composition of the VP including a distal meniscoid part and a proximal membranous part [5]. A first electron microscopic analysis of the fiber composition within the VP was published in 1994 [6]. The first real-time dynamic studies examining the VP with US were performed by the study group of Saito et al. in 2011 [16]. All studies represent a scientific challenge of exploring complex anatomical and biomechanical processes in small structures, some of which are in the sub-millimeter range.

In a clinical context, PIP joint distortion, pulley injuries, and VP avulsions are common. Most injuries are associated to direct trauma, and treatment strategies contain conservative and surgical regimes [8–11]. Different techniques for VP avulsion repair have been described [8–11]. However, the outcomes of these procedures are not always fully satisfactory and predictable [8–11]. Regarding a possible adaptation of conservative or reconstructive treatment techniques dependent on the VP injury pattern and location, our study results could be relevant in making physicians aware of the need to preserve both, the VP shape and VP position changes, to ensure full range of finger motion. This seems especially relevant in respect of preventing injury complications such as chronic boutonniere deformity. In patients with pulley rupture, previous studies have shown that A3 pulley damage and multiple pulley rupture can be detected indirectly by identifying a pathological VP shape [18]. According to our own experience with MRCF in a preliminary patient collective, the advantage of a dynamic VP evaluation might be that numerous flexion positions are available for a better identification of pathological VP shapes in cases of pulley rupture. Therefore, knowledge of the physiological full range of motion VP shape as presented in our study seems relevant for all treating physicians.

Our study has several limitations. We primarily investigated young male volunteers, and no further criteria such as race, comorbidities, or smoking status were analyzed. There may be changes noted to other populations. No accuracy assessment for MRI quantification methods was performed. Validations to proof that the claims based on the relatively low number of histological specimens accurately reflect what occurs in vivo are missing, especially as we did not perform true anatomical-MRCF comparison in the exact same ex vivo fingers. In addition, the age of the patients for histological evaluation did not exactly match the age group of the healthy volunteers. For reasons of practicality, our statistics pooled datasets of all individuals across 12 different bins, resulting in a somehow incremental analysis despite the dynamic nature of the study. For an even finer subgrouping, inclusion of considerably more datasets would have been necessary. Our analysis was focused on the midline sagittal PIP joint only with a relatively thick slice thickness of 2–3 mm, which was necessary to obtain sufficient signal-to-noise ratio. Despite the meticulous MRI study setup and histological preparation, we could not fully capture the three-dimensional VP shape or fiber courses inside the VP, especially not for the more lateral joint sections showing the check rein ligaments. In the future, full VP analysis including fiber course changes during motion would likely require much more extensive studies with technically innovative approaches (e.g., dynamic MRI fiber tracking). We encourage future studies to include new techniques such as MRCF in the evaluation of acute or chronic pathological conditions for a better prediction of postoperative outcome in clinical collectives.

In summary, this study is the first metric analysis of the physiological full range dynamic sagittal shape of the VP in real time using new MRCF techniques. This may contribute to a more comprehensive knowledge of the VP interaction with the PIP joint, which also could facilitate understanding of injuries and treatments in clinical cases such as pulley rupture or VP avulsion.

## Supplementary Information

Below is the link to the electronic supplementary material.

Supplementary file1 (MP4 2197 KB)
